# Observation of microstructure evolution during inertia friction welding using *in-situ* synchrotron X-ray diffraction

**DOI:** 10.1107/S1600577521001569

**Published:** 2021-03-19

**Authors:** Matthew Rowson, Chris J. Bennett, Mohammed A. Azeem, Oxana Magdysyuk, James Rouse, Ryan Lye, Joshua Davies, Simon Bray, Peter D. Lee

**Affiliations:** a Gas Turbine and Transmissions Research Centre, Faculty of Engineering, University of Nottingham, University Park, Nottingham NG7 2RD, United Kingdom; b School of Engineering, University of Leicester, Leicester LE1 7RH, United Kingdom; c Department of Mechanical Engineering, University College London, Torrington Place, London WC1E 7JE, United Kingdom; d Research Complex at Harwell, RAL, Didcot OX11 0FA, United Kingdom; e Rolls-Royce plc, PO Box 31, Derby DE24 8BJ, United Kingdom

**Keywords:** inertia friction welding, time-resolved synchrotron diffraction, phase transformation, non-equilibrium phase transformation

## Abstract

The widespread use and development of inertia friction welding is currently restricted by an incomplete understanding of the deformation mechanisms and microstructure evolution during the process. Understanding phase transformations and lattice strains during inertia friction welding is essential for the development of robust numerical models capable of determining optimized process parameters and reducing the requirement for costly experimental trials. A unique compact rig has been designed and used *in-situ* with a high-speed synchrotron X-ray diffraction instrument to investigate the microstructure evolution during inertia friction welding of a high-carbon steel (BS1407). At the contact interface, the transformation from ferrite to austenite was captured in great detail, allowing for analysis of the phase fractions during the process. Measurement of the thermal response of the weld reveals that the transformation to austenite occurs 230 °C below the equilibrium start tem­per­ature of 725 °C. It is concluded that the localization of large strains around the contact interface produced as the specimens deform assists this non-equilibrium phase transformation.

## Abstract 2

The first reported *in-situ* synchrotron X-ray diffraction experiments for the inertia friction welding process are presented. The evolution of the microstructure around the weld interface has been quantified throughout the process.

## 1. Introduction   

**Figure 1:**
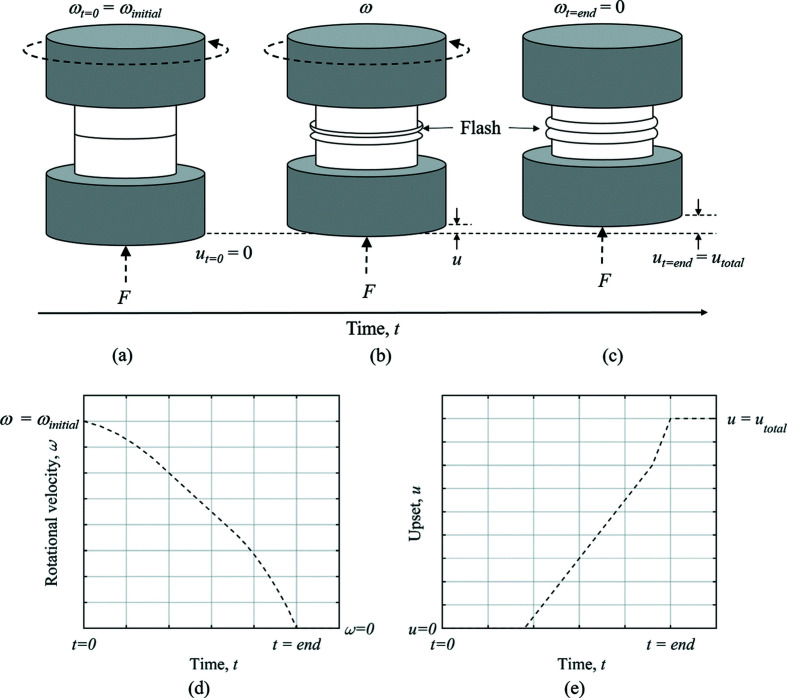
Schematic of the IFW process. (*a*) Workpieces brought into contact to start the process, generating heat at the weld interface due to friction at the contact interface. (*b*) Upsetting begins due to continued application of the axial load, where soft material around the weld interface is expelled as ‘flash’. (*c*) The end of the weld indicated by cessation of the rotational velocity. (*d*, *e*) The rundown in rotational velocity and upset (axial shortening), respectively, for clarification.

Inertia friction welding (IFW) is a solid-state joining process which is used to join axisymmetric components. One component is attached to a flywheel and rotated to a predefined initial rotational velocity (ω_initial_) to store energy for welding, while the other component is held stationary, as shown schematically in Fig. 1 ▸. The process starts when the drive is disengaged and the components are brought together under an axial load (*F*) applied to the stationary component. The relative rotational motion between the workpieces causes frictional heating at the contact interface, thereby softening the surrounding material which is expelled as ‘flash’ under the axial load. The expulsion of ‘flash’ causes axial shortening of the specimens, known as upset (*u*). As kinetic energy is converted to heat at the weld interface, the rotational velocity of the rotating specimen reduces throughout the process. The process is complete when the rotational velocity reaches zero. The two process outputs are the rundown in rotational velocity and the upset profile produced as the specimens shorten throughout the process.

The combination of initial rotational velocity (ω_initial_) and flywheel inertia (*I*) provide energy (*E*) for welding; however, in order to scale between weld geometries, this is better presented as specific energy (*S.E.*), as shown in equation (2), where *A* is the initial contact area between specimens,
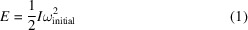


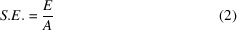
The combination of localized heating and pressure joins the materials. However, to produce a successful weld with complete bonding across the weld interface, a thorough understanding of the sensitivity of process response to variations in rotational velocity, flywheel inertia and axial pressure is required. After parametric investigation, IFW can be used to join high-strength materials, such as nickel-based superalloys (Chamanfar *et al.*, 2015 ▸), which can be difficult to weld using conventional fusion welding techniques. Furthermore, IFW is capable of welding materials with vast differences in strength, such as aluminium and steel (Taban *et al.*, 2010 ▸).

IFW is a complex process in which a combination of thermal, mechanical and microstructural effects dictate the evolution of the weld. To improve process understanding and the sensitivity of the process to input parameters, *ex-situ* investigations of IFW have utilized a range of methods to characterize the as-welded microstructure and mechanical properties for a range of materials, in both similar and dissimilar welds. Laboratory and synchrotron X-ray diffraction facilities have been used to characterize phase fractions of retained austenite produced by IFW of dissimilar high-strength steels Aermet100 and SCMV, concluding that the higher fraction of retained austenite on the SCMV specimen caused the increase in hardness in this workpiece (Moat *et al.*, 2008 ▸). Additionally, residual stresses in the as-welded condition and after heat treatment have been characterized in the axial and hoop directions by synchrotron diffraction for dissimilar steel welds (Moat *et al.*, 2009 ▸). This study also showed that post-weld heat treatment was much more effective on the Aermet100 workpiece. Scanning electron microscopy (SEM), transmission electron microscopy (TEM), *ex-situ* X-ray diffraction (XRD) and microhardness analyses have been used to quantify microstructure variation and correlate this to the mechanical response for as-welded and heat-treated IFWs of nickel-based superalloy RR1000 (Preuss *et al.*, 2002*a* ▸). Further analysis using *ex-situ* synchrotron and neutron dif­fraction methods quantified the difference in residual stresses for the as-welded and heat-treated IFWs, and suggested a modification to the heat-treatment tem­per­ature to further relax the residual stresses in the welds (Preuss *et al.*, 2002*b* ▸).

To assess the sensitivity of the process to the microstructure of the parent specimens, the as-welded microstructure and mechanical properties of IFWs of three different nickel-based superalloys were compared *via* synchrotron and neutron diffraction (Preuss *et al.*, 2006 ▸). Here, the weaker weld-line properties of alloys with low γ′ volume fractions were attributed to full dissolution and limited reprecipitation of the γ′ precipitates during and after IFW. The behaviour of RR1000 IFWs with different parent γ grain sizes were characterized by SEM and TEM, with the results showing a uniform region of fine recrystallized grains within the weld zone, regardless of parent grain size (Huang *et al.*, 2011 ▸). Analysis of residual stress profiles for both fine-grain and coarse-grain RR1000 IFWs also showed little variation with change in parent grain size (Iqbal *et al.*, 2011 ▸). Dissimilar IFW of nickel-based superalloys IN718 to RR1000 showed a reduction in strength at the weld interface due to the full dissolution of γ′ on the IN718 side (Daus *et al.*, 2007 ▸). This can be alleviated *via* post-weld heat treatment to produce welds with better mechanical properties than the parent materials. IFW of dissimilar superalloys LSHR and Mar-M247 has also been shown to produce weld joints of equal or greater strength than the parent materials, with a parametric investigation con­cluding that increased inertia provided fewer microstructural defects at the weld interface (Mahaffey *et al.*, 2016 ▸).

In recent years, finite element models have been developed for IFW as a means of predicting process outputs. Incorporation of effects such as interface friction behaviour and heat generation (Moal & Massoni, 1995 ▸), multibody contact and self-contact behaviour (D’Alvize *et al.*, 2002 ▸), and material definitions and phase transformations (Bennett *et al.*, 2013 ▸), have improved the accuracy of the outputs of these models. This has led to the production of models with the ability to predict process outputs (rundown and upset), tem­per­ature evolution and post-weld residual stresses. Whilst these modelling approaches can analyse the thermal and mechanical evolution of the process through representation of the process outputs, any microstructural predictions from these models can only be verified in the as-welded condition. To validate and develop microstructural predictions produced by numerical modelling methods, novel *in-situ* data is required to characterize deformation mechanisms and microstructure evolution. The development and production of numerical modelling approaches with the capability to predict all aspects of IFW will reduce the requirement of experimental weld trials which are costly in terms of both time and experimental resources.

Synchrotron X-ray beamlines facilitate the acquisition of *in-situ* microstructure evolution data. Synchrotron diffraction provides many benefits over conventional laboratory diffraction, such as increased X-ray beam voltage and smaller X-ray beam area. These benefits, coupled with the ability of high-acquisition-rate detectors, allow for the acquisition of *in-situ* diffraction data over extremely small time and length scales, which would not be possible using laboratory facilities.

*In-situ* time-resolved synchrotron X-ray diffraction has been utilized for gas tungsten arc (GTA) welding of steels (Wong *et al.*, 2003 ▸; Elmer, 2008 ▸) and titanium alloys (Elmer *et al.*, 2004 ▸), allowing for quantification of the phase transformations at various locations in the fusion zone and heat-affected zone (HAZ) during welding. These studies utilized beam voltages of 7–12 keV, beam diameters of 540–730 µm and detectors capable of acquiring diffraction data at 10–20 frames per second. However, due to the nature of IFW, where the weld interface moves as the specimens shorten, smaller X-ray gauge areas and higher diffraction acquisition rates are required. Recent work has shown the capability to characterize the microstructural variation across welded joints produced by various welding techniques and materials due to the increased beam voltage and reduced beam size available (Oliveira *et al.*, 2019 ▸, 2020 ▸). Beamline I12 of Diamond Light Source, UK, contains detectors capable of acquiring diffraction images at 450 frames per second at maximum resolution (Drakopoulos *et al.*, 2015 ▸). Additionally, the X-ray spectrum of 50–150 keV coupled with high flux allows for large penetration depths, thus facilitating the ability to conduct the desired experiments.

## 2. Methodology   

In order to conduct the *in-situ* diffraction experiments it was necessary to consider several features of the IFW process and modify them accordingly.

• Conventional IFW specimen geometries (>60 mm outer diameter) are too large for X-rays to penetrate. Therefore, the specimen geometry was proportionally scaled to 16 mm outer diameter and 12 mm inner diameter to permit acquisition of complete Debye–Scherrer diffraction rings at a rate of 100 fps.

• Reduction of the specimen geometry to the size used in this study required production of a bespoke small-scale IFW machine for microscale analyses (µ-IFW). The design of the µ-IFW apparatus had to adhere to a strict range of specifications to facilitate the success of the experiments. There could be no obstructions to the incident or diffracted X-rays, and size and weight (<200 kg) limitations were set to ensure safe mounting on synchrotron beamlines.

• A bespoke compact experimental setup was designed and built to allow for exploration of the complete processing window, through variation in the three process inputs (flywheel inertia, rotational velocity and axial pressure).

• The reduced specimen geometry was expected to increase the axial gradients of tem­per­ature and strain normally seen in the process. Thus, the axial variation in microstructure would likely occur over very small length scales. To resolve this variation in microstructure, an X-ray beam area of 100 µm × 100 µm was used. This beam area permitted analysis in the axial variation in microstructure whilst facilitating acquisition of complete Debye–Scherrer diffraction rings at the desired rate of 100 fps.

• To synchronize the weld process outputs recorded by the µ-IFW apparatus and the diffraction data acquired by the detector at Beamline I12, a voltage pulse was recorded by the µ-IFW data acquisition system with each image acquired.

• As the IFW specimens shortened during the weld, the weld interface moved relative to the synchrotron beam. Therefore, to monitor the microstructure at a single axial location throughout the process, repeat welds were conducted with the X-ray beam offset at various axial distances from the initial weld interface, allowing positions of interest (for example, the weld interface) to intersect the X-ray beam at different times during the weld as the specimens shortened.

### 2.1. Material and specimen geometry   

The material under investigation in this study was BS1407 steel, a high carbon tool steel which consists of a body-centred cubic (b.c.c.) ferritic microstructure at ambient tem­per­atures and a face-centred cubic (f.c.c.) austenite microstructure at elevated tem­per­atures. The composition of BS1407 was measured by spark emission testing and is presented alongside the typical composition in Table 1 ▸. The *Thermo-Calc* software (Andersson *et al.*, 2002 ▸) has been used to calculate the transformation tem­per­ature range for the ferrite to austenite transformation for this composition, which was predicted to occur between 725 °C and 734 °C.

**Figure 2:**
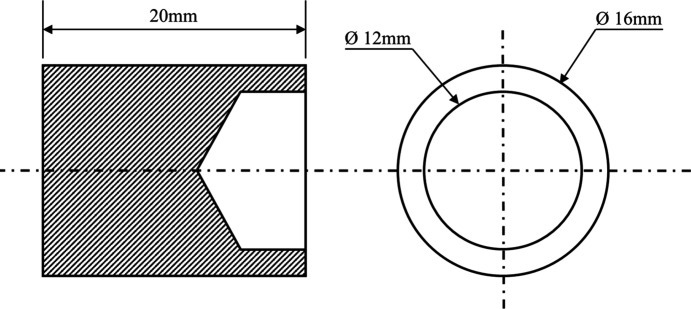
The dimensions of the IFW specimens used in the *in-situ* synchrotron diffraction experiments.

The weld specimen geometry was a 16 mm diameter cylinder with a length of 20 mm, as presented in Fig. 2 ▸. A 12 mm diameter hole was drilled to a depth of 4 mm on one face of the specimen to produce the weld interface feature, which provided a wall thickness of 2 mm for welding. This specimen geometry represents a typical weld geometry used in industry.

### 2.2. µ-IFW apparatus   

**Figure 3:**
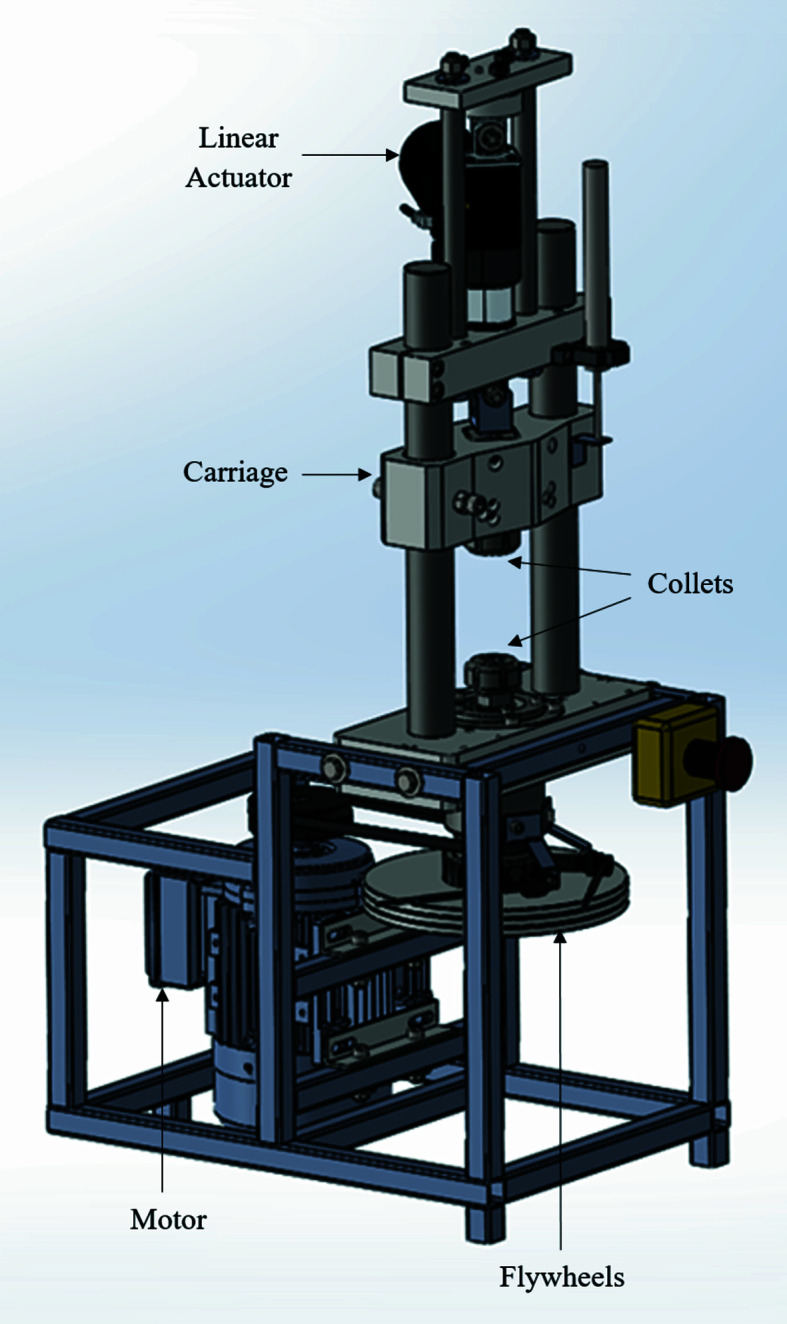
Annotated CAD-render of the µ-IFW apparatus produced to conduct the *in-situ* diffraction experiments.

The viability of these novel experiments relied on the ability to perform inertia friction welding with a sufficiently small sample geometry to allow for penetration of X-rays from the synchrotron source. A bespoke small-scale IFW machine (µ-IFW) was produced in order to conduct welds at the scale required, which is presented in Fig. 3 ▸. The weld specimens were held in collets throughout the process, and the collet nuts were tightened to a torque of 84 N m to ensure no axial or rotational slipping of the specimens occurred during welding. In the spindle assembly, the collet nut was threaded onto a shaft on which up to three flywheels, each providing 0.0176 kg m^2^ inertia, could be mounted. This shaft was driven by a three-phase motor, providing a rotational speed of up to 4140 rpm. This combination of rotational speed and flywheel inertia was able to produce a maximum of 4950 J of weld input energy, which provided a specific energy of 56.2 MJ m^−2^ for the specimen geometry used. In the fixture assembly, the collet was fitted to a carriage which was free to move axially. The rear side of this carriage was fixed to a linear actuator, which provided an axial load of up to 4000 N during the process. This load capacity allowed for weld pressures of 45.5 MPa to be applied with the specimen geometry utilized in these experiments.

To monitor process inputs and outputs, such as axial load, rotational velocity and upset throughout the process, a set of sensors were attached to the µ-IFW apparatus. The signals recorded by the sensors were processed by a National Instruments CompactDAQ and recorded *via* *LabView*. The *LabView* interface also provided remote control of the µ-IFW device.

The rundown in rotational velocity was measured using an RLS GTS35 gear tooth sensor (RLS, 2017 ▸). A gear wheel was attached to the spindle shaft and the sensor produced a voltage signal when a gear tooth passed it. This produced a square wave voltage signal, the frequency of which was converted to a rotational velocity. A TE FC23 compression load cell (TE Connectivity) was mounted to the rear of the linear actuator. When the actuator was engaged, the load cell was compressed, outputting the axial load applied by the actuator throughout the process. The upset was measured using an LVDT. This was mounted to the actuator mount and the ball tip rested on the carriage, thus providing the position of the carriage/fixture specimen throughout the process. The position data were then converted to upset data by taking the pre-weld pressure test position as zero upset and measuring the change from this point.

### 2.3. Beamline conditions   

Experimental Hutch 1 of Beamline I12 at Diamond Light Source was used for these experiments. The µ-IFW apparatus was mounted on the beamline sample stage which allows for translation in the *x*, *y* and *z* directions. This was used to align the synchrotron beam with the weld specimens and subsequently apply the axial offset of the beamline.

A Dectris Pilatus3 X CdTe 2M detector was used for acquisition of diffraction images. This detector had a resolution of 1475 × 1679 pixels with a pixel size of 172 µm × 172 µm, providing an extremely high resolution which results in sharp diffraction peaks for analysis. Additionally, the maximum acquisition rate of diffraction images with this detector was 250 fps. In this study, the acquisition rate was set to 100 fps, which allowed for acquisition of complete Debye–Scherrer diffraction rings for the material, X-ray beam energy and X-ray penetration depth. The sample-to-detector distance used throughout the study was 880 mm.

**Figure 4:**
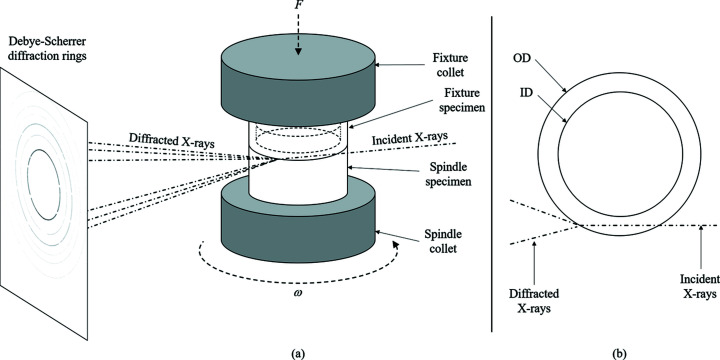
Schematic of the IFW setup, showing (*a*) an overview of the experimental methodology and (*b*) a plan view of the weld specimens to show the X-ray path in detail.

With the specimen geometry used to conduct the *in-situ* synchrotron diffraction experiments, axial gradients in tem­per­ature and strain during welding were expected to be extremely high, with mechanical deformation localized over a very narrow region about the weld interface. Therefore, to capture the microstructure evolution, and the axial gradient in this, a 100 µm × 100 µm monochromatic X-ray beam with 89 keV energy was used. The X-ray beam intersected a chord of the weld section, to minimize path length and prevent absorption of X-rays whilst ensuring the intersection of the X-ray beam and the centre of the specimen wall thickness. A schematic of this set-up is presented in Fig. 4 ▸.

A characteristic of IFW is the axial shortening of the specimens as they are forged together to complete the weld. As the axial load is applied on one specimen only, the contact interface moves during the process as the specimens shorten. Prior testing of the weld parameters indicated a total upset value approximately one order of magnitude larger than the X-ray beam height, and so the diffraction patterns for the microstructure at a static axial point could not be recorded throughout the process.

**Figure 5:**
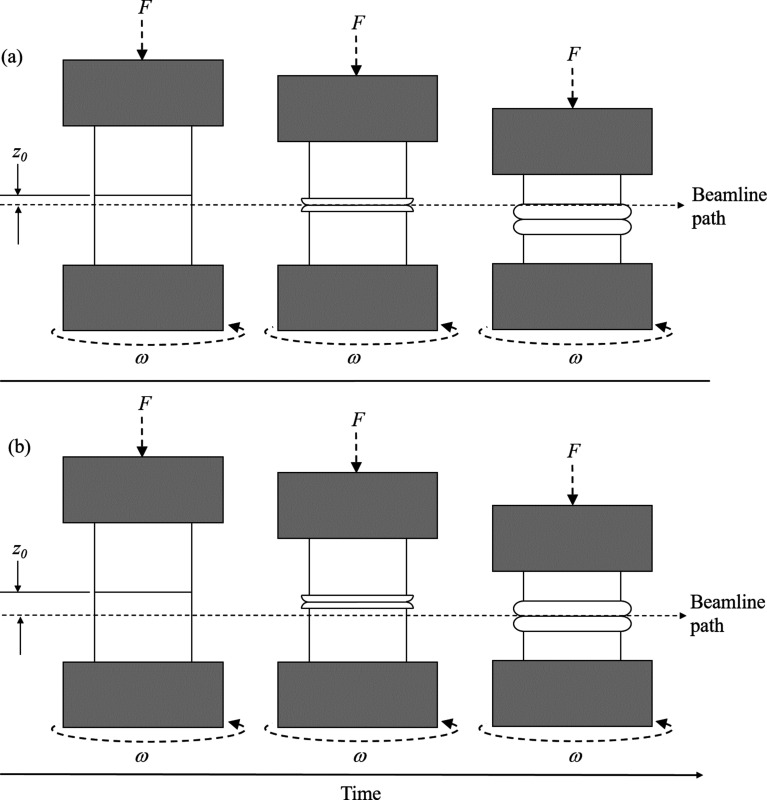
Schematic of the axial offset distance between the initial weld interface and the synchrotron beam. The offset allowed for diffraction data to be gathered from the weld interface at various time points during the weld: (*a*) a small axial offset caused an intersection of the weld interface and the synchrotron beam at the beginning of the weld, and (*b*) a larger axial offset required more deformation of the weld specimens to move the contact interface into the stationary beam, and so intersection between the weld interface and synchrotron beam did not occur until the end of the weld.

To allow for quantification of the microstructure at a single axial position (for example, the contact interface) throughout the process, several welds were performed with the same process parameters. For each weld, the axial position of the synchrotron beam was varied with respect to the initial contact interface, as shown in Fig. 5 ▸. The axial offset distance between the centre of the synchrotron beam and initial contact interface is denoted *z*_o_.

Using the contact interface as the point of interest, varying *z*_o_ provided the intersection of the contact interface and the beam centre at different time points during the weld. Increasing the value of *z*_o_ caused the intersection of the contact interface and synchrotron beam to occur later in the process.

### 2.4. Experimental methodology   

The weld parameters used in this study were: a rotational velocity (ω_initial_) of 4000 rpm, flywheel inertia (*I*) of 0.0344 kg m^2^ and an axial load (*F*) of 4000 N. For this set of input parameters, the five axial offset values used are presented in Table 2 ▸.

To provide quantitative diffraction data analysis, it was important to know the location of the weld interface relative to the stationary beamline throughout the process. To calculate the position of the weld interface throughout IFW, the assumption was made that both specimens deform symmetrically about the weld interface, allowing the axial distance of the weld interface relative to the centre of the beam area to be determined using the formula
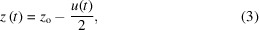
where *z*(*t*) is the position of the weld interface relative to the centre of the synchrotron beam area. *z*_o_ is the relative X-ray beam offset from the initial weld interface, as presented in Table 2 ▸, and *u*(*t*) is the weld upset. When *z*(*t*) is positive, the weld interface is positioned above the synchrotron beam centre, and negative values indicate that the weld interface is below the synchrotron beam. This relationship allowed the diffraction images when the synchrotron beam was aligned with a point of interest to be determined.

### 2.5. Data analysis   

**Figure 6:**
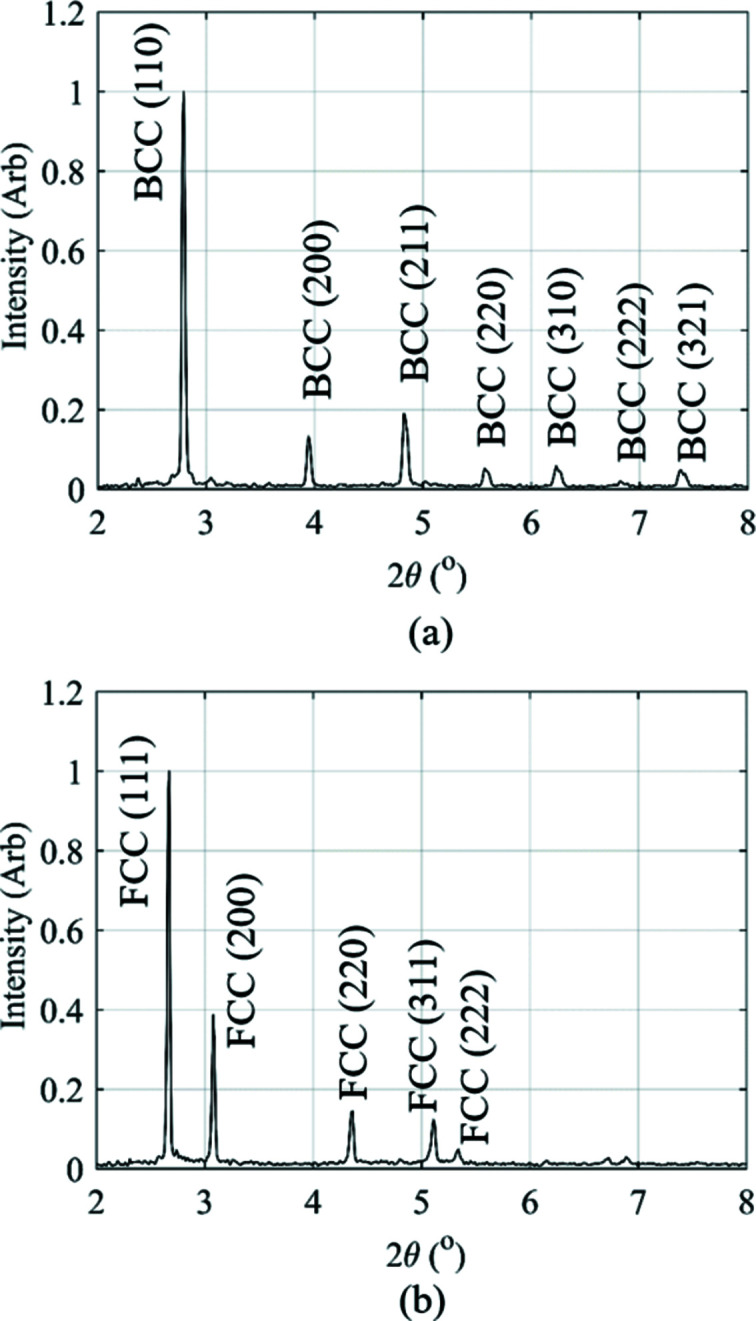
Diffraction patterns produced by the 360° azimuthal integration for (*a*) the b.c.c. ferrite microstructure in the parent material and (*b*) the f.c.c. austenite produced during IFW.

The *Data Analysis WorkbeNch* (*DAWN*) software (Basham *et al.*, 2015 ▸; Filik *et al.*, 2017 ▸) was used for azimuthal integration of the Debye–Scherrer diffraction rings. To calibrate synchrotron instrument parameters, ceria (CeO_2_) was used. Fig. 6 ▸ presents the diffraction patterns observed in the parent material, where a fully ferritic microstructure is present, and during the weld when only the austenite phase exists in the microstructure.

The crystallographic data analysis software *GSAS-II* (Toby & Von Dreele, 2013 ▸) was used to perform a full Rietveld analysis and estimate the phase fractions of b.c.c. ferrite and f.c.c. austenite during IFW. For this analysis, only the b.c.c. ferrite and f.c.c. austenite phases were included in the refinement, as negligible phases such as carbides and graphite were not of interest in this study. Furthermore, the texture parameters were not refined during this analysis, as that is beyond the scope of the analysis presented here.

**Figure 7:**
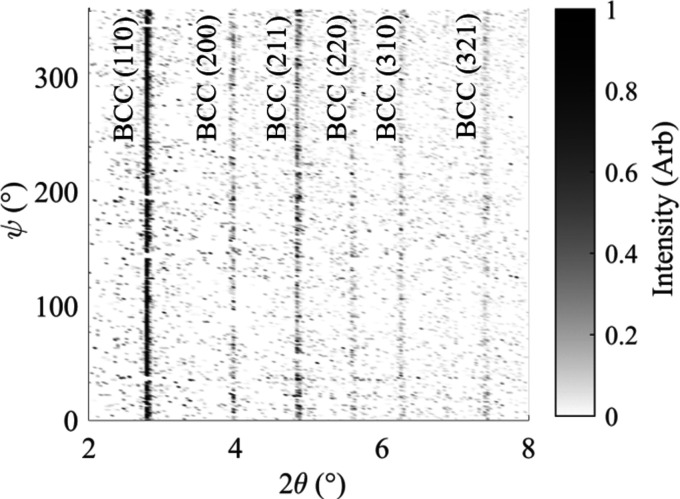
The diffraction pattern of b.c.c. ferrite at 1° intervals in the azimuth range.

To ensure that full azimuthal integration was feasible, the variation in the diffraction pattern with the azimuth angle in the diffraction rings has been investigated. Using a pre-weld diffraction image of b.c.c. ferrite recorded from weld P4 at *t* = 0 s, the diffraction rings have been divided into bins of 1° in the azimuth and plotted for the full 360° rings. This is presented in Fig. 7 ▸.

It can be seen in Fig. 7 ▸ that there is very little variation in the diffraction angle of the b.c.c. ferrite peaks across the 360° ψ range. This shows that there is minimal directional effect in the material prior to welding, and full azimuthal integration can be performed for these images. It should be noted that the noise present in Fig. 7 ▸ is attributed to the high acquisition rate and high detector sensitivity.

**Figure 8:**
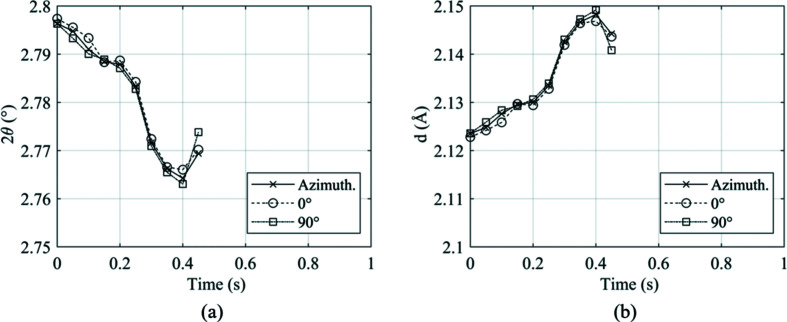
Variation in peak parameters throughout weld P4 of the (110) b.c.c. ferrite peak for (*a*) the diffraction angle 2θ and (*b*) the *d*-spacing.

However, the study presented in Fig. 7 ▸ only accounts for one image recorded prior to welding. Therefore, to ensure that any directional effects did not become prominent during the whole welding process, images were extracted at 0.05 s increments from weld P4. Weld P4 was chosen for this analysis as both ferrite and austenite were present for a significant portion of the weld duration. For each diffraction image, 10° slices at the principal axes 0 and 90° ψ were produced, alongside full Azimuthal integration, producing three unique diffraction patterns. For each diffraction pattern, the b.c.c. (110) and f.c.c. (111) peaks, when present, were fitted. The variation in 2θ and *d*-spacing for each peak throughout the duration of welding was recorded and is presented in Fig. 8 ▸ for the b.c.c. (110) peak.

Fig. 8 ▸ shows that despite there being fluctuations in the peak values during the process, there is little difference in the value shown by full azimuthal integration when compared with the principal angles. When comparing against the azimuthal integration, there was no more than 0.16% difference between the value measured in either principal direction, for either variable.

**Figure 9:**
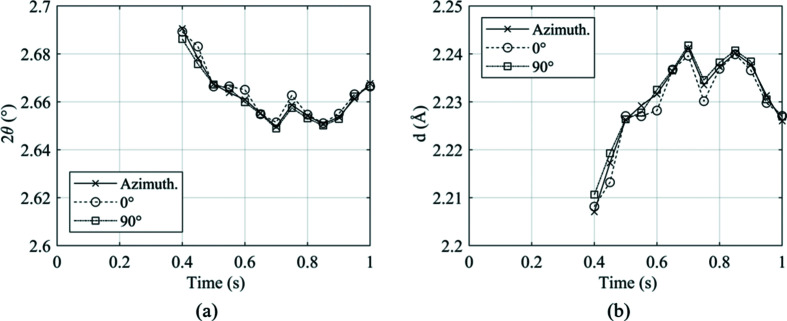
Variation in peak parameters throughout weld P4 of the (111) f.c.c. austenite peak for (*a*) the diffraction angle 2θ and (*b*) the *d*-spacing.

Fig. 9 ▸ presents the variation in peak parameters throughout weld P4 for the austenitic f.c.c. (111) peak. Similar to the b.c.c. (110) peak, there is little variation between the principal directions and the full azimuthal integration values. The maximum difference measured was between the 0° direction and full integration at *t* = 0.6 s, with a difference of 0.15%.

## 3. Results   

### 3.1. IFW outputs   

**Figure 10:**
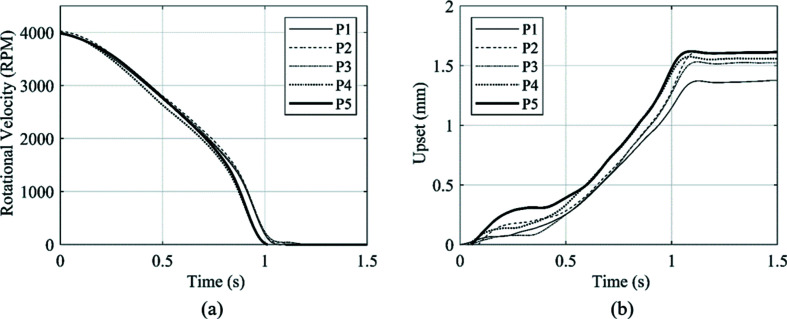
Weld outputs for the five repeat welds, showing (*a*) the rundown in rotational velocity and (*b*) the weld upset.

The rotational velocity and upset of the five repeat welds conducted are presented in Fig. 10 ▸ to provide an assessment of the repeatability of the process with the developed experimental system.

The rundown curves in Fig. 10 ▸(*a*) show a mean weld duration of 1.14 s. The standard deviation of the five welds was 0.010 s, which is 8.76% of the mean value. The upset curves presented in Fig. 10 ▸(*b*) provided a mean total upset value of 1.54 mm. Over the five repeat welds, the standard deviation was 0.087 mm, which accounts for 5.66% of the mean value. The standard deviation of the repeat welds was within 10% of the mean value for both measured weld outputs, and therefore, the welds can be treated as equivalent for the purpose of this study.

**Figure 11:**
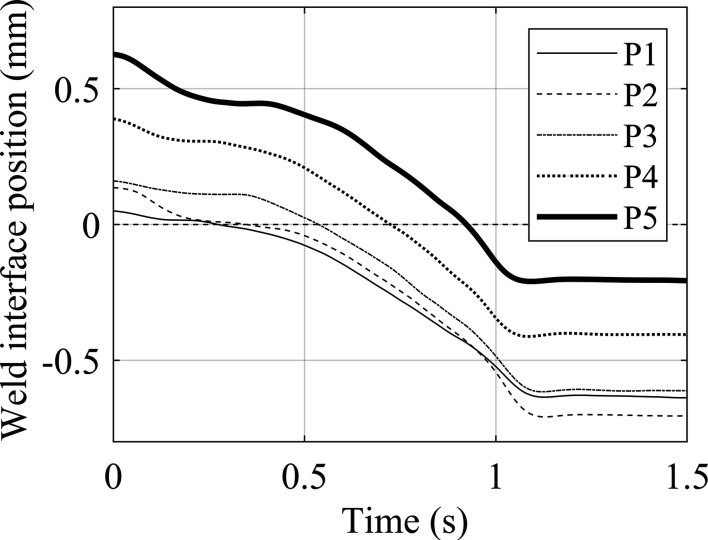
Weld interface position relative to the synchrotron beam centre for the five welds conducted.

For each weld, equation (3) has been used to convert the upset data to the position of the weld interface relative to the centre of the synchrotron beam. This data is shown in Fig. 11 ▸. For each individual weld, the weld interface intersected the centre of the synchrotron beam at a different time. Note that the contact interface positions at the start of each weld, *t* = 0 s, were consistent with the axial offset positions presented in Table 2 ▸.

### 3.2. X-ray diffraction data   

#### 3.2.1. Time-resolved XRD spectra   

**Figure 12:**
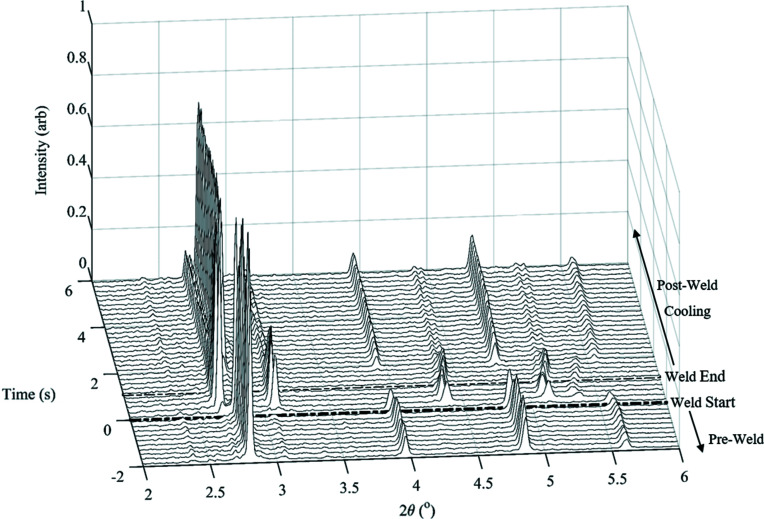
Waterfall plot of the XRD data series produced during weld P1.

Fig. 12 ▸ presents a series of time-resolved XRD patterns recorded during weld P1, where every 20th image of the 100 fps data is plotted for clarity. The pre-weld parent microstructure shown in the XRD patterns is that of b.c.c. ferrite. The parts came into contact and the weld commenced at *t* = 0 s. Within the first 0.2 s of the weld, the parent ferrite started to transform to f.c.c. austenite.

Once the weld was completed at approximately 1.1 s, no further heat generation occurred in the weld and the effects of cooling can be seen. The reverse transformation from austenite to ferrite began at approximately 1.6 s. The intensity of the b.c.c. ferrite peaks increased between 1.6 and 3 s, and remained constant after 3 s. After cooling, the weld consisted of a two-phase microstructure, as both b.c.c. ferrite and f.c.c. austenite peaks were present with no variation in intensity.

#### 3.2.2. Process-resolved XRD spectra   

**Figure 13:**
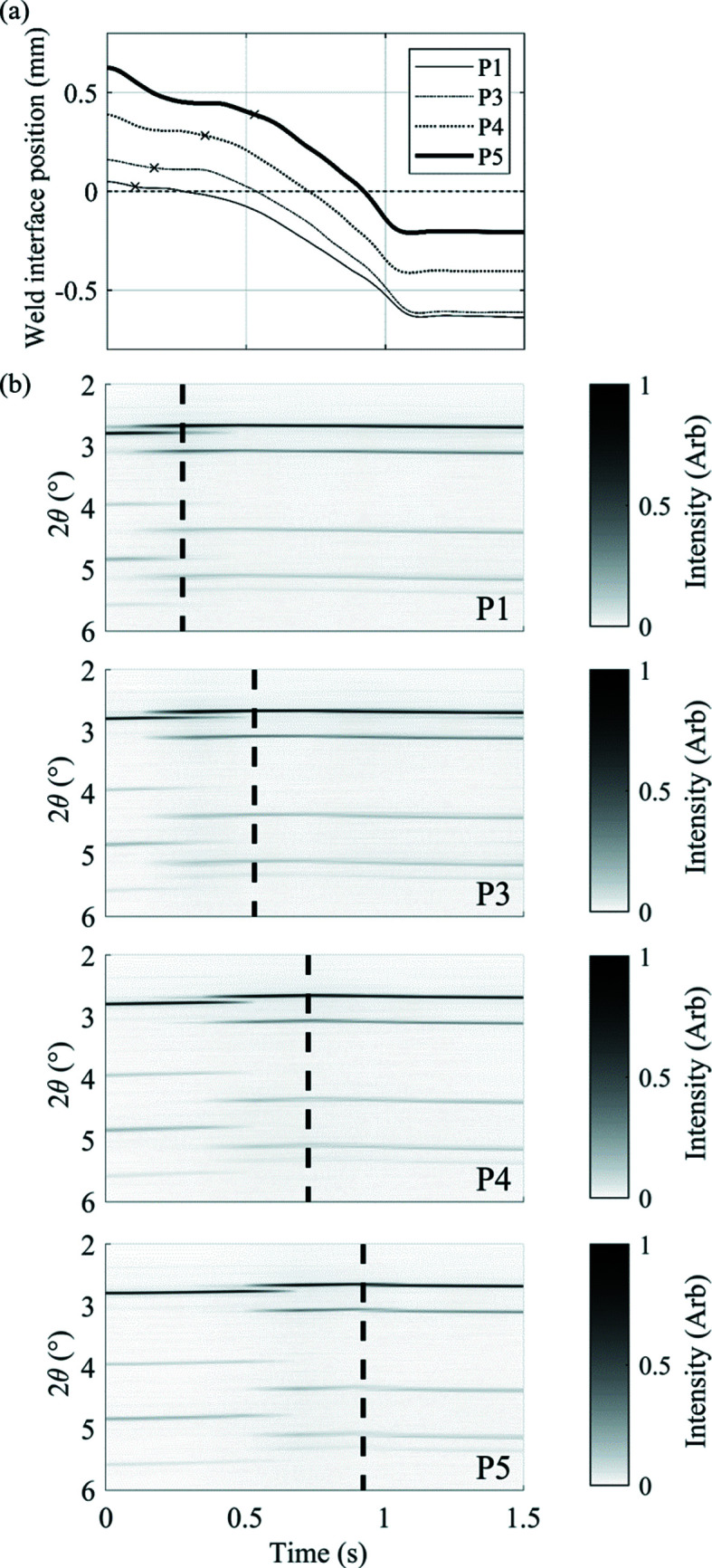
Comparison of the weld interface position and the diffraction data series for four welds, showing (*a*) the position of the weld interface relative to the beamline centre and (*b*) the X-ray diffraction data series labelled for repeat welds. The vertical dashed line in each plot of part (*b*) represents the intersection of the synchrotron beam centre and the weld interface presented in part (*a*). Each position curve in part (*a*) is marked with a cross at the time at which the transformation from ferrite to austenite occurs in the respective series of diffraction data, showing the increasing size of the HAZ during welding.

Through comparison of the raw diffraction data acquired for each weld, some initial trends in the results can be seen. Here, weld P2 has been removed due to the small variation in *z_o_* between this weld and the neighbouring welds. Fig. 13 ▸ presents the weld interface positions for the welds alongside the XRD data series for each individual weld.

In each series of diffraction data, the transformation from b.c.c. ferrite to f.c.c. austenite can be seen. Comparing welds shows that by increasing the axial offset (*z*_o_) value, the time taken for this transformation to occur increased. This is due to the larger amount of upset required to bring the region near the weld interface, where tem­per­atures are highest, into the beamline.

During IFW, heat is generated at the weld interface and subsequently conducted axially along the weld specimens. This causes the production of a heat affected zone (HAZ), which is commonly defined as the region in which the tem­per­ature is high enough to cause changes to the microstructure. Therefore, in each weld presented in Fig. 13 ▸, the presence of austenite in the diffraction data was expected to occur when the HAZ enters the beamline. For each successive weld, the value of *z*(*t*) at which the ferrite-to-austenite transformation begins has been highlighted on the plot of beamline positions in Fig. 13 ▸(*a*). The size of the HAZ is shown to increase throughout the weld, due to generation and conduction of heat throughout the process, as welds with a larger *z*_o_ values display the presence of austenite in the microstructure at larger *z*(*t*) values.

#### 3.2.3. Quantification of austenite volume fraction   

**Figure 14:**
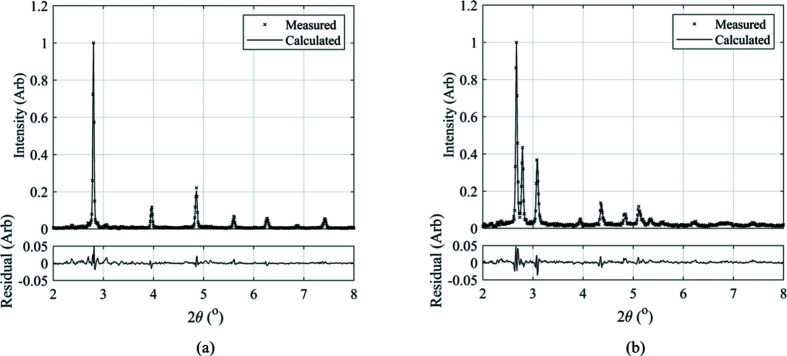
*GSAS-II* refinement for diffraction images (*a*) for the parent material prior to welding and (*b*) at the weld interface 0.28 s into the weld.

Fig. 14 ▸ presents the *GSAS-II* Rietveld analysis of two diffraction patterns produced during weld P1. The diffraction image recorded prior to welding shown in Fig. 14 ▸(*a*) consists of b.c.c. ferrite with a lattice parameter of 3.0038 Å and a phase fraction of 99.8%. At 0.28 s, when the synchrotron beam centre was aligned with the weld interface, the phase fractions are calculated to be 30.2% austenite and 59.8% ferrite. The lattice parameter of f.c.c. austenite here is 3.8431 Å.

**Figure 15:**
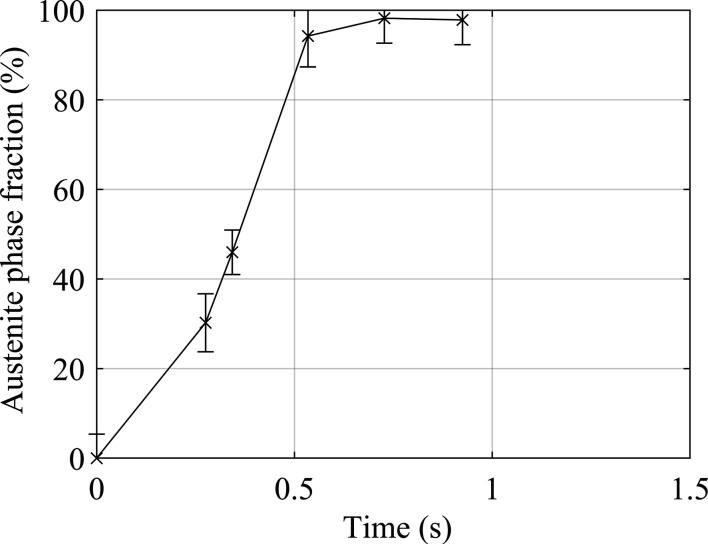
Evolution of the phase fraction of austenite at the weld interface during IFW. Each data point is from a different weld, refined from the diffraction image taken when the weld interface and the synchrotron beam centre were coincident. The point at *t* = 0 s was evaluated from a pre-weld diffraction image of the parent microstructure.

For each weld, the diffraction image acquired when the weld interface was aligned with the centre of the synchrotron beam has been extracted and full Rietveld analysis has been performed to estimate the phase fractions in each image. This allows for quantification of the austenite phase fraction at the weld interface throughout IFW and is presented in Fig. 15 ▸.

The microstructure at the weld interface transformed from fully ferritic at the start of the weld to an austenite fraction of 30.2% at a time of 0.28 s. The formation of austenite in this time suggests initial heating rates in excess of 2000 °C s^−1^. After a weld time of 0.5 s, the weld interface austenite phase fraction was in excess of 95%; however, at no point during the weld did the austenite fraction reach 100% as expected. It is thought that the high acquisition rate used in the experiments has increased the contribution of background noise in the diffraction patterns. Due to this, there still appeared to be a small [110] b.c.c. peak of similar lattice parameter to the ferrite phase, which contributed up to a 5% fraction of ferrite.

### 3.3. *Ex-situ* thermal validation of diffraction data   

**Figure 16:**
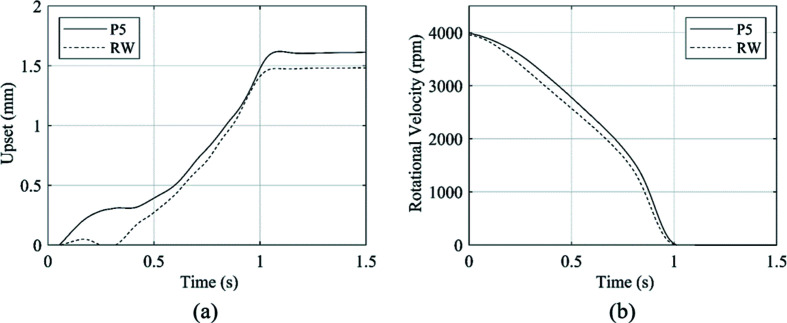
Comparison of weld output data for XRD weld P5 and the repeat weld (RW) with *ex-situ* thermocouple measurement, showing (*a*) upset and (*b*) rundown.

To validate the phase evolution seen in the processed dif­fraction data, weld P5 was repeated with a spot-welded type K thermocouple attached to the fixture specimen. This thermocouple had a sensitivity of 41 µV °C^−1^, with an uncertainty of ±2.2 °C at ambient tem­per­ature and ±7.5 °C at 1000 °C. The position of the thermocouple was consistent with the *z*_o_ value used for the *in-situ* XRD weld. Again, the repeatability of the weld outputs was checked to ensure that the welds were similar, and the assumption can be made that the tem­per­ature profile was consistent with the XRD data. These data are shown in Fig. 16 ▸.

For the repeat weld, the weld duration and total upset had variations of −0.960 and −8.19%, respectively, from weld P5. Additionally, the profiles of the two rundown curves are consistent, which indicates that the energy input rates and thus the heat input rates were similar. Therefore, it is logical to assume that the thermal validation of the XRD data is accurate within stated errors.

**Figure 17:**
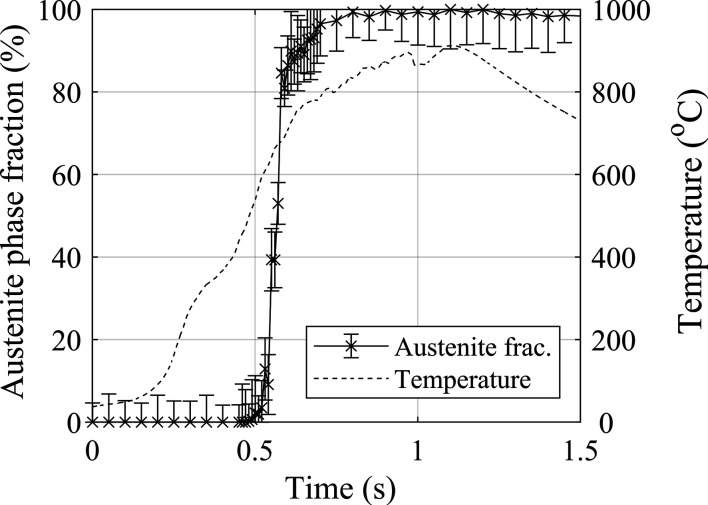
Austenite phase fraction data from weld P5 aligned with the thermal data from the repeat weld. The error bars are defined by the residual of the Rietveld refinement.

Fig. 17 ▸ presents the austenite volume fraction recorded from weld P5 alongside the tem­per­ature measurements from the repeat weld. The error in the austenite fraction is evaluated as the residual value produced by the Rietveld refinement method. The austenite phase fraction increased above the value of the parent microstructure at 0.48 s. At this time, the tem­per­ature in the repeat weld was 495 °C, which is 230 °C lower than the start tem­per­ature of the ferrite-to-austenite transformation calculated by *Thermo-Calc*.

The results clearly show that the transformation from ferrite to austenite occurred below the equilibrium transformation tem­per­ature; however, there is a combination of thermal and mechanical effects which occur during IFW which can assist the occurrence of non-equilibrium phase transformations. To investigate this, the approach proposed by Ramesh & Melkote for predicting white layer formation during high-speed machining of tool steels has been used (Ramesh & Melkote, 2008 ▸; Duan *et al.*, 2013 ▸). This method estimates the tem­per­ature of a phase transformation based on the stress and strain energy which the material is subjected to, as follows,
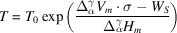
where *T* is the transformation tem­per­ature which occurs under a stress σ and strain energy *W_S_*. *T*_0_ is the equilibrium transformation tem­per­ature, 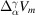 is the molar volume change, and 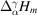 is the molar enthalpy of the transformation from α (ferrite) to γ (austenite).

The tem­per­ature data recorded from the repeat weld were used as the transformation tem­per­ature, *T*. The stress, σ, was calculated from the axial pressure from the weld. Equation (4) was then rearranged to calculate the strain energy required to cause the transformation from ferrite to austenite at the stress and tem­per­ature recorded in the weld. Using stress–strain data for BS1407, the strain energy was converted into a strain value for comparison with the experimental data.

**Figure 18:**
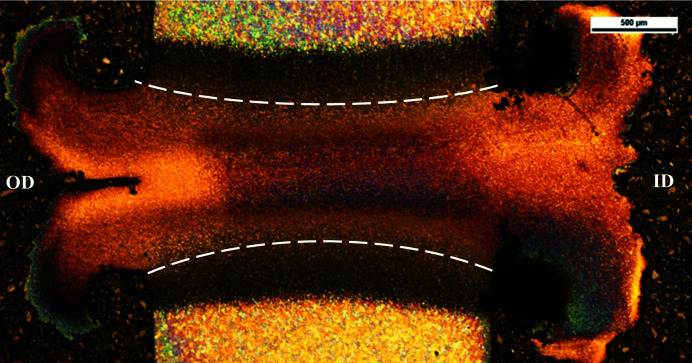
An optical micrograph of weld P5. The white lines show the deformed zone at the weld interface.

**Figure 19:**
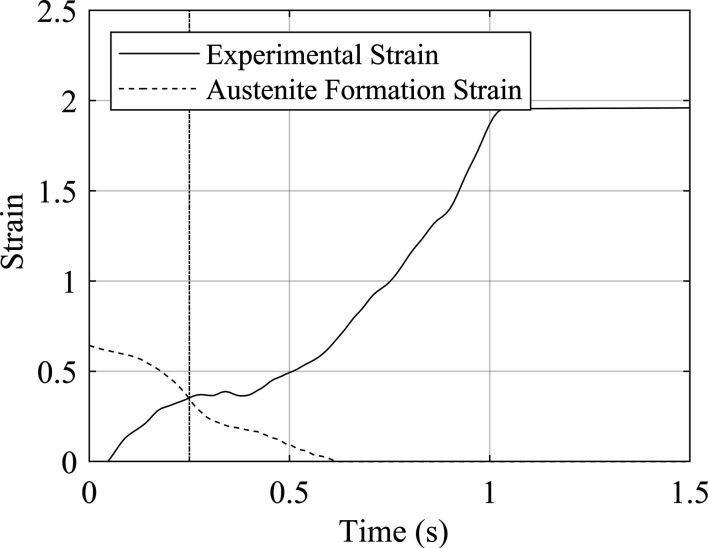
Estimated experimental strain and the strain required to form austenite calculated from tem­per­atures recorded during the repeat weld. The vertical line at *t* = 0.25 s indicates the point at which these lines intersect.

To estimate the strain which occurs during the process, the size of the deformed zone of a radially cross-sectioned weld has been measured using optical microscopy, as shown in Fig. 18 ▸. From this, the minimum and maximum thickness were determined as 0.76 mm and 1.04 mm, respectively. Across the weld specimen chord which the beamline intersects, the average deformed zone thickness was calculated to be 0.86 mm. Assuming the total upset occurred over the deformed zone, the experimental strain was estimated as follows,
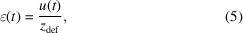
where ɛ(*t*) is the strain at time *t*, *u*(*t*) is the upset at time *t* and *z*_def_ is the mean axial length of the deformed zone. The comparison between the experimental strain data and the strain required to form austenite at reduced tem­per­atures is shown in Fig. 19 ▸. This suggests that the magnitude of the strain in the deformed zone was large enough to assist the formation of non-equilibrium austenite at a weld time of 0.25 s, which is earlier than the time at which austenite is seen in the diffraction data of weld P5.

To verify the estimated experimental strain profile, the variation of ferrite and austenite lattice parameters have been analysed to estimate the microstrain during the process. First, the parent lattice parameters were calculated. For ferrite, this has been taken from pre-weld data, producing a value of 3.0038 Å. As the austenite forms during the process, tem­per­ature and strain have an influence on the lattice parameter. Therefore, post-weld data has been analysed for weld P1, as the position of the beamline at the end of the weld is furthest from the weld interface, and so the effects of tem­per­ature and strain on the lattice parameter will be least significant. The average value of the austenite lattice parameter in the post-weld state was 3.7747 Å.

To correctly understand the evolution of the lattice parameters of both phases present, the effects of tem­per­ature and strain must be deconvoluted. To do so, reference data for the lattice parameters of ferrite and austenite at difference tem­per­atures (Yu *et al.*, 2018 ▸) have been normalized and applied to the parent lattice parameters found in this experiment, to produce relationships between the lattice parameters and tem­per­ature. These relationships have been applied to the tem­per­ature data presented in Fig. 17 ▸ to estimate the ferrite and austenite lattice parameters produced at the tem­per­atures experienced in this weld. The microstrain was calculated using 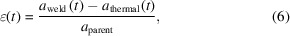
where ɛ(*t*) is the strain in the phase, α_weld_ is the lattice parameter of the phase recorded during IFW, α_thermal_ is the estimated lattice parameter of the phase at the recorded tem­per­ature and α_parent_ is the lattice parameter of the parent material. Here, α_weld_ is calculated from the mean lattice parameter derived from the peak position of the four peaks with lowest diffraction angle (b.c.c. ferrite: [110], [200], [211], [220]; f.c.c. austenite: [111], [200], [220], [311]).

**Figure 20:**
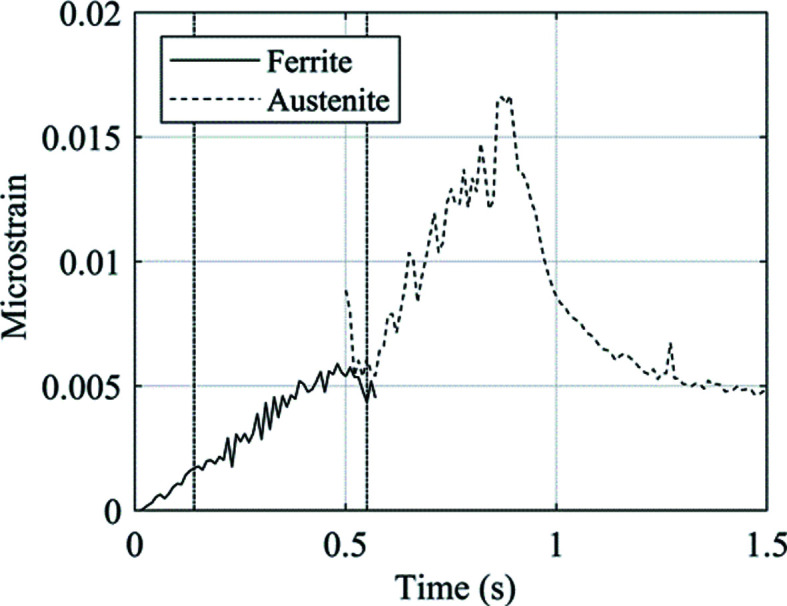
The microstrain calculated from the lattice parameter evolution of ferrite and austenite. The vertical dashed lines show the time points at which the centre of the X-ray beamline intersects the outer and inner bounds of the deformed zone.

The strain of both the ferrite and austenite phases is presented in Fig. 20 ▸, alongside the time at which the centre of the X-ray beamline intersects the outer and inner bounds of the deformed zone. This experimental microstrain data matches the trends of the estimated macrostrain, and so there is confidence in the assumptions made.

**Figure 21:**
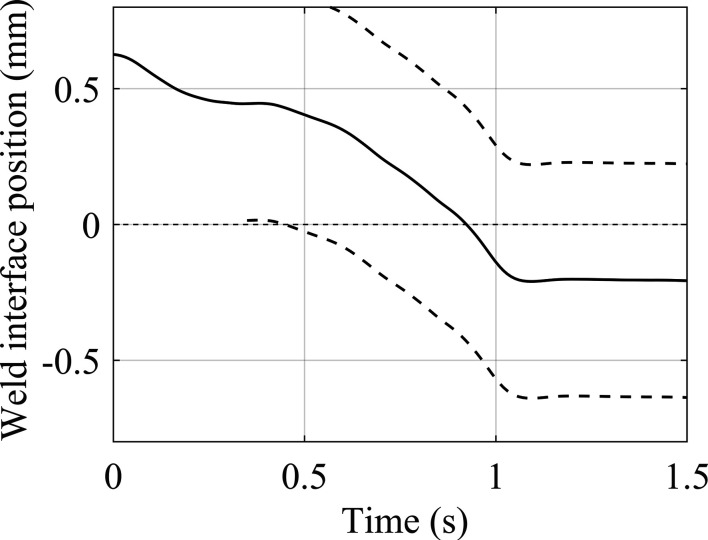
The position of the weld interface relative to the synchrotron beam for weld P5. The dashed lines show the average thickness of the deformed zone from a time of 0.35 s.

The approach used has estimated that the strain produced in the deformed zone was high enough to assist the formation of non-equilibrium austenite at a weld time of 0.25 s. However, austenite was not present in the diffraction data until a weld time of 0.48 s. To fully understand this, the position of the weld interface relative to the synchrotron beamline throughout the process must be considered. This is presented in Fig. 21 ▸. In addition, position data at ±0.43 mm from the weld interface is presented to show the size of the deformed zone. The size of the deformed zone is presented from a weld time of 0.35 s onwards, as this coincides with the onset of steady-state deformation, as seen in Fig. 16 ▸(*a*). Prior to the occurrence of mechanical deformation, it is assumed that the deformed zone developed in size and that during steady-state deformation the deformed zone size remained constant due to the linear energy input rate and upset rate. It can be seen that the mean deformed zone length intersects the centre of the synchrotron beam at 0.46 s.

Utilizing reasonable assumptions, it has been estimated that the strain present in the deformed zone of this weld was large enough to assist the formation of non-equilibrium austenite at a weld time of 0.25 s. As the weld progressed, the deformed zone was pushed into the stationary X-ray beamline at 0.46 s, which in turn displayed the presence of austenite in the diffraction data at 0.48 s.

## 4. Concluding remarks   

The use of synchrotron diffraction at the Diamond Light Source for *in-situ* microstructural observation during the IFW process has been presented. Time-resolved XRD patterns have been refined in order to show the transformation from ferrite to austenite on heating, and the reverse transformation on cooling. The latter transformation is much slower due to the air quenching experienced by the weld; however, it has been discovered that cooling rates after IFW are sufficiently high to retain a significant fraction of austenite in the microstructure.

Five welds have been performed in order to quantify the phase composition at the weld interface of BS1407 steel IFWs at five separate time points during the process by increasing the offset distance between the X-ray beam and the initial weld interface. This process has shown the transformation from ferrite in the pre-weld microstructure to a fully austenitic microstructure at the weld interface within 0.52 s of the weld commencing. Furthermore, the 52% phase fraction of austenite found at the weld interface 0.27 s into the weld suggests that heating rates at the interface are in excess of 2000 °C s^−1^.

Thermal measurements have presented a possible source of the occurrence of the non-equilibrium phase transformation in the deformed zone during IFW. Use of beamline location monitoring, deformed zone size calculation and estimation of the strain required to produce austenite in thermally dominated conditions provide a possible explanation for why thermal and diffraction data are inconsistent in this case.

This novel methodology has provided a quantifiable analysis of the microstructure of weld specimens during the IFW process. Whilst this study has focused only on the weld interface, the same methodology can be repeated for the range of locations measured throughout the experiments in order to quantify the microstructure throughout the near-weld region.

## Figures and Tables

**Table 1:** Elemental composition of BS1407 steel used in these experiments

Element	Fe	C	Cr	Mn	Si	Other
Spark emission testing (%)	97.5	1.21	0.413	0.35	0.22	0.307
BS1407 Typical composition (%) (T&A Precision, 2021 ▸)	97.818	1.13	0.43	0.37	0.22	0.032

**Table 2:** The five axial offset distances between the centre of the synchrotron beam and the initial weld interface used during the repeat welds

Position name	Axial offset from initial weld line, *z*_o_ (mm)
P1	0.05
P2	0.1
P3	0.15
P4	0.375
P5	0.6
